# Muscle dysmorphia: Could it be classified as an addiction to body image?

**DOI:** 10.1556/JBA.3.2014.001

**Published:** 2014-02-03

**Authors:** ANDREW C. FOSTER, GILLIAN W. SHORTER, MARK D. GRIFFITHS

**Affiliations:** ^1^School of Experimental Psychology, University of BristolBristolUK; ^2^Bamford Centre for Mental Health and Wellbeing, University of UlsterLondonderryUK; ^3^MRC All Ireland Trials Methodology Hub, University of UlsterLondonderryUK; ^4^International Gaming Research Unit, Division of Psychology, Nottingham Trent UniversityNottinghamUK; ^1^School of Experimental Psychology, University of BristolBristolUK; ^2^Bamford Centre for Mental Health and Wellbeing, University of UlsterLondonderryUK; ^3^MRC All Ireland Trials Methodology Hub, University of UlsterLondonderryUK; ^4^International Gaming Research Unit, Division of Psychology, Nottingham Trent UniversityNottinghamUK

**Keywords:** muscle dysmorphia, behavioral addiction, body dysmorphic disorder, body image, obsessive–compulsive disorder, eating disorder

## Abstract

**Background:**

Muscle dysmorphia (MD) describes a condition characterised by a misconstrued body image in which individuals who interpret their body size as both small or weak even though they may look normal or highly muscular. MD has been conceptualized as a type of body dysmorphic disorder, an eating disorder, and obsessive–compulsive disorder symptomatology.

**Method and aim:**

Through a review of the most salient literature on MD, this paper proposes an alternative classification of MD – the ‘Addiction to Body Image’ (ABI) model – using Griffiths (2005) addiction components model as the framework in which to define MD as an addiction.

**Results:**

It is argued the addictive activity in MD is the *maintaining of body image* via a number of different activities such as bodybuilding, exercise, eating certain foods, taking specific drugs (e.g., anabolic steroids), shopping for certain foods, food supplements, and the use or purchase of physical exercise accessories). In the ABI model, the perception of the positive effects on the self-body image is accounted for as a critical aspect of the MD condition (rather than addiction to exercise or certain types of eating disorder).

**Conclusions:**

Based on empirical evidence to date, it is proposed that MD could be re-classified as an addiction due to the individual continuing to engage in maintenance behaviours that may cause long-term harm.

## INTRODUCTION

Muscle dysmorphia (MD) describes a condition characterised by a misconstrued body image in which individuals interpret their body size as both small and weak even though they may look normal or even be highly muscular (Pope et al., 2005). Those experiencing the condition typically strive for maximum fat loss and maximum muscular build. MD can have potentially negative effects on thought processes including depressive states, suicidal thoughts, and in extreme cases suicide attempts (Pope et al., 2005). These negative psychological states have also been linked with concurrent use of Appearance and Performance Enhancing Drugs (APED) including Anabolic Androgenic Steroids (AAS) (Mosley, 2009; Pope et al., 2005). The use of these substances may not just relate to body image, but also social or sexual aspects such as producing an enhanced libido or a sense of physical and psychological wellbeing (Cohen, Collins, Darkes & Gwartney, 2007).

MD was originally categorised by Pope, Katz and Hudson (1993) as Reverse Anorexia Nervosa, due to characteristic symptoms in relation to body size. It has been considered to be part of the spectrum of Body Dysmorphic Disorders (BDD); one of a range of conditions that tap into issues surrounding body image and eating behaviours (McFarland & Karninski, 2008). Parallels have also been drawn with Obsessive–Compulsive Disorder (OCD) given some similarities in symptom expression like ritualistic activity (Phillips, 1998). Consequently, there is a lack of consensus amongst researchers whether MD is a form of BDD, OCD or a type of eating disorder (e.g. Jones & Morgan, 2010; Maida & Armstrong, 2005; Murray, Rieger, Touyz & De la Garza Garcia, 2010; Nieuwoudt, Zhou, Coutts & Booker, 2012; Pope, Gruber, Choi, Olivardia & Phillips, 1997; Pope et al., 2005). In this paper, the limitations of these classification approaches will be discussed, and an alternative model is proposed – the ‘Addiction to Body Image’ (ABI) model.

## HOW IS MUSCLE DYSMORPHIA CURRENTLY CLASSIFIED?

BDD is characterised by a preoccupation with a perceived defect in physical experience that leads to a substantial functional impairments (American Psychiatric Association, 2013). Such a definition can include MD and in the latest DSM-5, muscle dysmorphia was added as a specifier to the BDD diagnostic criteria. This representation of Muscle Dysmorphia is supported by authors such as Pope et al. (1997). In the context of a preoccupation with the belief that their body is not sufficiently muscular and lean, and excessive attention to exercise, lifting weights and diet (possibly including supplements and AAS), the criteria outlined by Pope et al. (1997) – for which two or more need to be present for a diagnosis of the condition – are:

1. Giving up important activities of a social, work or recreational nature due to a strong need to maintain activities in relation to workouts and diet control.
2. Active avoidance of situations where their body is displayed to others, and an intense distress/anxiety of these situations when they are unavoidable.
3. Clinically significant distress arising from pre-occupation with their body fat, size, or musculature.
4. A continuation of dietary control and exercise, despitethe knowledge of adverse physical or psychological consequences.

The International Classification of Diseases (ICD-10) also classifies MD with other BDD conditions in section F45.2 entitled hypochondriacal disorder. Essential features include somatic complaints, preoccupation, and distress in relation to physical appearance. The category appears to refer to a heterogeneous range of conditions, and the somatoform description of the MD condition appears unwarranted. Somatoform disorders relate to physical symptomatology that is difficult to explain in terms of physical disease, substance use, or other mental disorder. Mosley (2009) considered the ‘somatoform’ description incongruent with MD; Maida and Armstrong (2005) concurred, given MD symptoms were found to be unrelated to symptoms of somatoform disorder in men who regularly lifted weights.

Other classifications consider MD to be part of the obsessive–compulsive disorder symptomatology. A shift of BDDs to be classified as OCD spectrum disorders was considered but rejected due to a lack of evidence (Phillips & Hollander, 1996). There are similarities in symptom expression including intrusive fear, ritualistic actions or obsessions in the course of the illness (Bienvenu et al., 2000; Phillips, 1998; Phillips, Dwight & McElroy, 1998; Phillips, Gunderson, Mallya, McElroy & Carter, 1998; Rosen, Reiter & Orosan, 1995; Zimmerman & Mattia, 1998). Despite overlaps with symptoms and comorbid conditions, Phillips, Gunderson et al. (1998) note important disparities in social isolation, delusions, and differences in insight that cast doubt on MD’s suitability for classification on the OCD spectrum.

There are also some parallels drawn to the eating disorders such as anorexia nervosa or bulimia nervosa given the extent of attention to diet and exercise, and dissatisfaction with body image (Mangweth et al., 2001; Olivardia, Pope, Mangweth & Hudson, 1995). Eating disorders as presented in the Diagnostic and StatisticalManual are characterised by severe disturbances in eating behaviour and a preoccupation with eating (American Psychiatric Association, 2013). The rigour in which an individual pursues the body ideal is similar amongst the different types of eating disorder and MD. However, the goals being pursued are very different (e.g. the intrusive fears around weight relate to gain in Anorexia Nervosa, but loss in MD). Additionally, it could be considered that a secondary feature of the MD condition is the disordered eating (Olivardia, 2001), and thus classification as a disorder of ‘eating’ is not sufficient. Other authors (e.g., Demetrovics & Griffiths, 2012) have mentioned that MD could perhaps be classed as an addiction although there was limited explanation.

## AN ALTERNATIVE CLASSIFICATION: ‘ADDICTION TO BODY IMAGE’ MODEL

The ‘Addiction to Body Image’ (ABI) model attempts to provide an operational definition and to introduce a standard assessment across the research area. The ABI model uses the addiction components model of Griffiths (2005) as the framework in which to define muscle dysmorphia as an addiction. For the purposes of this paper, body image is defined as a person’s “perceptions, thoughts and feelings about his or her body” (Grogan, 2008, p. 3). The addictive activity is the *maintaining of body image* via a number of different activities such as bodybuilding, exercise, eating certain foods, taking specific drugs (e.g., anabolic steroids), shopping for certain foods, food supplements, and purchase or use of physical exercise accessories). Addiction is defined as the use of a substance or activity that becomes all-encompassing to the user and comprises all six of Griffiths’ (2005) addiction components. Each of these components is described below in the context of MD symptomatology and behavioural maintenance.

### Salience

A person with an ABI may: (i) have cognitive disturbances that lead to a total preoccupation with activities that maintain body image such as physical training and eating according to a strict dietary intake (Veale, 2004), (ii) be able to perform other tasks such as work and shopping (explained by reverse salience – see below) as these tasks will be designed and built around being able to engage in specific body image maintenance behaviours such as physical exercising and eating (Olivardia, Pope & Hudson, 2000), and (iii) be able to manipulate their personal situation to ensure they can perform these maintenance tasks (Mosley, 2009). The individual with ABI may even change or forego career opportunities and other daily activities as it may reduce their ability to train or control eating behaviour during the day (Murray et al., 2010).

### Reverse salience

If the person with ABI cannot engage in maintenance behaviours such as training or eating regimes, their thought processes are likely to be excessively preoccupied by the need to carry out the desired behaviours to maintain body image (Olivardia et al., 2000). This may result in the manifestation of physical symptoms. More specifically, the cognitive disturbance creates a negative thought process that facilitates the manifestation of physical symptoms (e.g., shakes, sweating, nausea, etc.) as seen in other addictions. Due to some of the dietary restrictions the person with ABI places upon their body, physical symptoms such as fainting and falling unconscious may be present due to low blood sugar levels.

### Mood modification

For an individual with ABI, being able to engage in the maintenance behaviours brings a sense of reward. As a consequence, training and food intake (either restrictive or over-eating) should facilitate the release of endorphins into the bloodstream, which would increase positive mood. The physical act of engaging in physical exercise and training (whether cardio- or weight-based) may produce a physical state whereby the muscles are enriched with blood (which at their biggest is known as a ‘pump’). This pump brings a sense of euphoria and happiness to the person (Elliot, Goldberg, Watts & Orwoll, 1983).

The ABI model proposes that engaging in the maintenance behaviours – for example weight training – will create a chemical high created by the body though the release of chemicals such as endorphins (Griffiths, 1997). A person with ABI will desire these chemical changes and this may have the same effect (both physiologically and psychologically) as other psychoactive substances. Once their maintenance behaviours have been completed, the person’s mood will relax due to completion of the activity, and the person may also have a feeling of utopia, a sense of inner peace, or an exceptional high. This feeling has been linked to the use of AASs in gym training (Mosley, 2009).

The person with ABI will need to control their food intake (i.e., less or more protein and carbohydrates). The ABI model proposes this will become a secondary dependence due to the food intake being part of the process to maintain the primary dependence (i.e., the sculpting of the body). This will be due to the body adapting to the amount of calories it is being fed, but also due to requirement of being lighter or heavier – and for longer – which in turn will allow the person to obtain the desired body shape.

### Tolerance

The person with ABI may need to increase the levels and intensity of the training or the food restriction (i.e., the maintenance behaviours) to achieve the desired physiological and/or psychological effects. This can be achieved through different training strategies or by the consumption of different foods. In some circumstances, this may be achieved through the use of psychoactive substances such as AASs or other food inhibiting drugs. Record keeping of training sessions and seeking out changes in activities may assist the individual in combatting the effects of tolerance (Mosley, 2009).

### Withdrawal

The person with ABI is expected to have negative physical and/or psychological effects if they are unable to engage in the maintenance activities. This would be likely to include one or more psychological and/or physical components (Griffiths, 2005) such as intense moodiness and irritability, anxiety, depression, nausea, and stomach cramps. They will not be able to just stop the maintenance behaviours without experiencing one or more of these symptoms.

### Conflict

The person with ABI becomes focused on their maintenance behaviours of training and/or eating. These behaviours can become all consuming, and the need to train, control diet, and exercise may conflict with their family, their work, the use of resources (e.g., money) and their life in general. An individual quoted in Mosley (2009) noted “bodybuilding is my life, so I make sacrifices elsewhere” (p. 194). In some cases of the addiction, the process is thought have healthy physical consequences and add to life in the short-term, in the long-term, the addiction will detract from their overall quality of life.

### Relapse

If the person with ABI manages to stop the maintenance behaviours for a period of time, they may be susceptible to triggers to re-engage in the behaviours again. CBT approaches for treatment of MD include aspects which address triggers or reinforcing behaviour, and reducing stress around maintaining body image to prevent likelihood of relapse (Grieve, Truba & Bowersox, 2009). When a person with ABI re-engages with behaviours again, they may go straight back into previous destructive training and eating patterns.

The ABI model differs from other addiction models in relation to the primary and secondary dependencies. For instance, in exercise addiction, the individual has the primary goal of exercising, and the cognitive dysfunction in this condition is the act of exercising in, and of, itself (Berczik et al., 2012). If the person loses weight or increases their body size through their exercise, this is seen as a secondary dependence as it is a natural consequence of the primary dependence and is not the primary goal. In MD, the primary dependence is maintenance in behaviours that facilitate body size change due to the cognitive dysfunction of negative perceptions of their body image. Exercise and/or dietary controls are the secondary dependence as they assist in achieving the primary goal of maintaining their desired body size and composition. In addition, exercise addiction tends to relate to compulsive aerobic exercise, with the endorphin rush from the physical exertion rather than a reward from physique change. Pope et al. (1997) also note that (to a degree) aerobic exercise may be avoided by those with MD as it may reduce muscle size.

In the ABI model, the perception of the positive effects on the self-body image is accounted for as a critical aspect of the MD condition. The maintenance behaviours of those with ABI may include healthy changes to diet or increases in exercise. However, such behaviours can hide or mislead those with ABI away from the negative thought processes that are driving their addiction. It is in the cognitive dysfunction of MD where we believe there is a pathological issue, and why the field has encountered problems with the criteria for the condition. The attempt to explain MD in the same manner as other BDDs may not be adequate due to the cognitive dysfunction occurring in the context of the potentially positive physical effects via improvements in shape, tone, and/or health of the body.

The ABI model supports the findings of Pope et al. (2005); there is a difference in the cognitive dysfunction with a misconstrued self-body image compared to other BDDs. The cognitive dysfunction causes the individual with MD to have a misconstrued view of their own body image, and the person may believe they are small and puny. This negative mindset has the potential to cause depression and other disorders, and may facilitate the addiction. Unlike other conceptualizations of MD in the BDD literature, we would argue that the agent of the addiction is the perceived body image that is maintained by engaging in secondary behaviours such as specific types of physical activity and food. The most important thing in the life of someone with MD is how their body looks (i.e., their body image). The behaviours that the person with MD engages in (such as excessive exercise or disordered eating) are merely the vehicles by which their addiction (i.e., their perceived body image) is maintained.

Based on empirical evidence to date, we propose that Muscle Dysmorphia could be re-classified as an addiction due to the individual continuing to engage in maintenance behaviours that cause long-term psychological damage. More research is needed to explore the possibilities of MD as an addiction, and how this particular addiction is linked to substance use and other comorbid health conditions. Controversy about the conceptual measurement of the condition, has led to a number of different scalesadapted from different criteria that may not fully measure the experience of MD (Cafri & Thompson, 2007). However, a group of questions that might test the applicability of the ABI approach to measuring and conceptualising MD have not been asked. Questionnaires such as the Exercise Addiction Inventory (Griffiths, Szabo & Terry, 2005; Terry, Szabo & Griffiths, 2004) and the Bergen Work Addiction Scale (Andreassen, Griffiths, Hetland & Pallesen, 2012) could be adapted to fit MD characteristics. Adequate conceptualisation is key to explore the clinically relevant condition (Kuennen & Waldron, 2007). This new ABI approach may also have implications for diagnostic systems around similar conditions such as other BDDs or eating disorders. Theoretical and empirical work exploring these in an addiction context would be welcomed.

